# Awareness, Practice, and Barriers Regarding Cervical Cancer Screening Among Women of Kageshwori Manohara Municipality, Nepal

**DOI:** 10.1155/ogi/5325540

**Published:** 2025-05-19

**Authors:** Pratima Pathak, Pratima Ghimire, Shyam Kala Chaudhary, Nebina Piya, Nira Shrestha

**Affiliations:** ^1^Department of Nursing, Maharajgunj Nursing Campus, Institute of Medicine, Tribhuvan University, Kathmandu, Nepal; ^2^Department of Nursing, Nepal Medical College, Kathmandu University, Kathmandu, Nepal; ^3^Department of Nursing, Nepalese Army Institute of Health Sciences, Tribhuvan University, Kathmandu, Nepal

## Abstract

**Background:** Cervical cancer remains a global public health concern occurring in most cases in developing countries. Furthermore, it is a highly preventable disease; it remains to be the most common cancer among Nepalese women. Assessing women's awareness of cervical cancer and identifying barriers to screening are of vital importance for the utilization of cervical cancer screening. Therefore, this study has been conducted to assess information regarding awareness, practice, and barriers to cervical cancer screening among the women of Kageshwori Manohara Municipality of Kathmandu district in Nepal.

**Methods:** Community-based descriptive cross-sectional study was conducted among 249 women aged 30–60 years by using a systematic random sampling technique. Data were collected from 1st to 29th February 2024 through a self-constructed semistructured tool using a face-to-face interview technique. Descriptive statistics and the chi-square tests were used for statistical analysis.

**Results:** Out of the total respondents, only 10.4% had adequate awareness regarding cervical cancer screening and 38.6% of the respondents had ever been screened for cervical cancer. Among the respondents who had ever been screened, the majority (86.5%) of their last time screening was within 5 years. A statistically significant association was found between levels of awareness with the age of the respondents (*p*=0.031), educational level (*p*=0.013), and number of children (*p*=0.003). However, no significant association was found with other variables such as age at marriage, ethnicity, occupation, and monthly family income. Absence of symptoms (54.6%), unaware of screening (17.7%), and feeling of embarrassment (11.6%) were the most mentioned barriers to practicing cervical cancer screening among the respondents.

**Conclusion:** The result of this study showed most of the respondents had an inadequate level of awareness and low experience of practicing cervical cancer screening. Therefore, community-based awareness campaigns and screening health camps should be conducted to increase knowledge and practice of cervical cancer screening.

## 1. Introduction

Cervical cancer ranks as the fourth leading cause of cancer in women in the world, having affected 604,000 women with over 341,000 deaths in 2020 [1]. Of these, about 90% occur in low- and middle-income countries, and if extensive actions are not taken, deaths are expected to increase by 25% over the next decade [2, 3]. Cervical cancer is the first most frequent cancer among women, particularly affecting those between 15 and 44 years of age, in Nepal [4, 5]. Every year, 2332 women are diagnosed with the disease, and 1367 die from the disease in Nepal [4]. Evidence shows that cervical neoplasia is etiologically related to human papillomavirus (HPV) infection transmitted through sexual intercourse [6]. The disease is largely preventable with a complete strategy of efficient screening, treatment, and a preventative vaccine [7].

As per the global strategy of the World Health Organization developed to expedite the elimination of cervical cancer, up to 70% of all women should undergo screening for cervical cancer using a high-performance test at least twice in their lifetime, once at age 35 and again at age 45, to reduce the number of deaths from the disease [8]. The WHO guidelines developed in 2021 recommended an organized system for screening, diagnosis, treatment, and follow-up of cervical cancer. The guideline stated that the primary mode of screening must involve the detection of HPV DNA, whereas cytology-based screening (Pap test) and visual inspection with acetic acid (VIA) are other forms of diagnostic tool [2]. To reach the objectives regarding widespread screening, existing barriers have to be identified and overcome [9]. In low- and middle-income countries, the most frequently mentioned barriers include a lack of awareness about risk factors and prevention, cultural and religious influences, and social stigmas associated with discussing reproductive health issues. These factors minimize women's insight into cervical cancer and its prevention, especially among younger groups, thus limiting the benefits [10].

In Nepal, the government has recognized screening for cervical cancer, followed by treatment when needed, as a basic right of all women. As a result, in 2010, national guidelines for cervical cancer screening were developed to achieve a 50% screening rate among women aged 30–60 years by the year 2015. Accordingly, the national guidelines recommended VIA as the primary screening tool at every level of care from primary to tertiary [5]. However, in 2015, only 5.4% of women aged 30–65 years and 8.2% of women aged 30–49 years in 2019 were ever screened for cervical cancer [11].

The effectiveness of cervical cancer screening is closely related to the knowledge and awareness of the disease among women. Hence, understanding the barriers to screening is also an important key to improving participation. There have been meager studies conducted regarding the assessment of awareness, practices, and barriers concerning cervical cancer screening in the community setting among Nepalese women. Thus, this study has been carried out to assess information regarding the awareness, practices, and barriers of cervical cancer screening among women aged 30–60.

## 2. Methods

### 2.1. Study Design and Setting and Sample Size

A community-based descriptive cross-sectional study was carried out in Kageshwori Manohara Municipality which lies in Kathmandu District in the Bagmati Province of Nepal consisting of 16 wards. Among the total 16 wards of the municipality, Ward No. 2, Aalapot was selected in regards to the convenience of the researcher. The sample size was estimated by using the formula for definite proportion, i.e., *n*=*Z*^2^pq/*d*^2^ with the assumptions of 5% allowable error, 95% confidence interval, 10% nonresponse rate, and 17.9% prevalence (*p*=0.179). The prevalence of adequate knowledge was taken from the community-based cross-sectional study conducted in Budhanilkantha Municipality of Kathmandu district [12]. Hence, the required sample size was 249.

### 2.2. Study Population and Sampling

Women aged 30–60 years living in Aalapot-2 since last six months had given their permission to take part in the study were chosen. The 30–60 age range was included in this study since it is the target age range for screening specified in the 2010 National Guidelines on Cervical Cancer Screening and Prevention in Nepal. Women who previously had a hysterectomy or cervical cancer were not included.

As per the records of the municipality office, the total number of households present during the study time was 752. Of the total 752 households, 249 households were chosen using a systematic random sampling technique. The sampling interval was calculated as 752/249 = 3.02. To elaborate further, the study population was recruited by going to every third household and identifying every woman within 30–60 years of age within that family. Only one eligible woman was selected from each household. Where there was more than one eligible woman, a simple random technique (lottery method) selecting one participant was used. If there was not an eligible woman in the third household, the team advanced to the fourth household or the fifth until an eligible woman was recruited.

### 2.3. Study Tools

The tool has been prepared based on the literature review and consulting with subject experts. The tool was divided into four sections. The first section consists of questions related to sociodemographic information. The second consists of questions related to awareness of cervical cancer screening which includes meaning, risk factors, symptoms, preventive measures, and screening of cervical cancer. There were a total of 10 questions. Scoring of “1” was given for each correct response and “0” for the wrong response. Some items were in multiple-response forms, whereas some were in multiple-choice forms that resulted in a total of 30 scores. If the total score of ≥ 50% (≥ 15 correct responses) was considered as adequate awareness and the score of < 50% (< 15 correct responses were regarded as inadequate awareness). The score classification has been adopted from similar studies conducted in various studies [13, 14].

The third section consists of questions related to the women's practice regarding cervical cancer screening utilization which was assessed in terms of having “ever” vs. “never” had screening, frequency, and the last testing. Practices regarding cervical cancer screening were expressed in terms of frequency and percentage. The fourth section consists of questions in which women who did not have cervical cancer screening were asked the reasons perceived by them that hindered cervical cancer screening. The question included possible options for barriers with multiple choices including other options. The respondent's response was filled in the additional options specified as others if their reason for not being screened was incompatible with the specified options.

### 2.4. Data Collection

Face-to-face interview technique was taken at the participant's house using a Nepal questionnaire from February 1, 2024, to February 29, 2024. The interview was conducted in a private space to maintain the course of the interview and avoid interruptions from other family members. The approximate time to collect data from each respondent was 20–25 min. Pretesting was conducted in 10% of the total sample size in a similar setting, i.e., among 25 women residing outside the study area and accordingly, necessary modifications were made.

### 2.5. Data Analysis

For data analysis, the data were entered into EpiData Version 3.1 and exported to Statistical Package for Social Science (SPSS) Version 16. Descriptive statistics such as frequency, percentage, mean, and standard deviation were used to determine sociodemographic data, awareness level, practice, and barriers associated with cervical cancer screening. The chi-square test was used to see whether certain independent variables and awareness level of cervical cancer screening were associated. *p* values below 0.05 were regarded as statistically significant.

### 2.6. Ethical Consideration

Ethical approval to carry out the study was taken from the “Institutional Review Committee” (reference number: 48-080/081) of the Nepal Medical College. Data were collected after granting written permission for data collection from the ward office of Ward No. 2 of Kageshwori Manohara Municipality. Similarly, respondents were assured that their information would be kept confidential, and verbal informed consent was taken from each of them.

## 3. Results

Out of a total of 249 respondents, 39.4% were in between the age group of 30–39 years with mean ± (SD) age being 42.05 ± (8.37) years. Similarly, most of the respondents, i.e., 94.4% were married and 42% had married within the age group of 15–19 years with mean ± SD (19.88 ± 3.743). Likewise, 50.2% of the respondents belonged to the Janjati ethnicity, and 31.7% of the respondents had completed a secondary level of education. In the same way, half of the respondents (50.2%) stated household work as their occupation. Similarly, among the 240 respondents who stated that they had at least one child, 77.5% had 1-2 numbers of children. Concerning average monthly family income, 75.1% responded their income was ≤ 40,000 (Table 1).

Among the total respondents, 41.8% correctly answered the meaning of cervical cancer. Likewise, one-third of the respondents (33.3%) responded poor perineal hygiene as one of the risk factors of cervical cancer, while 37.3% of the respondents were not aware of any one of the risk factors. Likewise, almost half of the respondents (48.6%) stated lower abdominal pain as one of the signs and symptoms of cervical cancer, and 36.5% of the respondents were not aware of any of the signs and symptoms (Table 2).

The majority of the respondents (80.3%) answered cervical cancer is a preventable disease. In response to preventive measures for cervical cancer, 35.3% responded maintaining perineal hygiene as one of the measures. The majority (79.1%) of the respondents had not heard about the HPV vaccine and only a few respondents (12.9%) answered correctly the appropriate time for the HPV vaccine. Most of the respondents (71.5%) were not aware of the available screening methods, while 16.9% of the respondents answered Pap smear as one of the screening methods. Similarly, 29.7% of the respondents answered correctly the ideal time to start cervical cancer screening. Only a few respondents (9.6%) answered correctly about the time interval for cervical cancer screening, and the majority (86.7%) of respondents' stated cervical cancer is curable if diagnosed in its earliest stage (Table 3).

Out of the total respondents, very few respondents that are only 10.4% had adequate awareness regarding cervical cancer (Table 4).

Of the total respondents, 38.6% of the respondents have been screened at least once for cervical cancer. Among the total 96 respondents who have ever been screened, the majority (86.5%) of their last time screening was within 5 years. Nearly half (46.9%) of the respondents had screened for cervical cancer at a health camp. Likewise, 38.5% responded Pap smear was used in screening, while 32.3% were unaware of the methods used in screening. Similarly, 37.5% of the respondents stated the reason for performing the test was following suggestions from health personnel. Nearly half (44.8%) stated they would go for screening only if gynecological problems occur. Out of a total of 153 respondents who had never been screened, the top reason for barriers to screening was because of no presence of symptoms (54.6%). However, the least number of the respondents (0.8%) responded to barriers to cervical cancer screening as due to lack of time (Table 5).

The findings showed there is a statistically significant association between levels of awareness with the age of the respondents (*p*=0.031), educational level (*p*=0.013), and number of children (*p*=0.003). However, no significant association was found with other variables such as age at marriage, ethnicity, occupation, and monthly family income (Table 6).

## 4. Discussion

The result of this study shows that only 10.4% of the respondents had adequate awareness regarding cervical cancer screening. Similar findings have been observed in similar studies conducted in the midwestern part of Nepal [14] and in Kathmandu [15], Nepal, where 13% and 11.3% of the respondents, respectively, had adequate knowledge of cervical cancer screening. Slightly higher findings have been observed in studies conducted by Devkota et al. (17.9%) [12] and Ampofo et al. (18%) [16]. Likewise, much higher findings have been observed in studies conducted by Getachew et al. (27.7%) [17], Bharati et al. (47.8%) [18], and Shrestha and Dhakal (34.4%) [19]. This difference in the proportion of the participants having adequate awareness levels might be explained due to the difference in study time and geographical variation and could be due to factors such as cultural and societal factors, education level, and healthcare infrastructures and also to slight differences in the definition between studies.

Among the total respondents, 41.8% correctly answered the meaning of cervical cancer in this study which is comparable to the findings conducted in Kathmandu, Nepal (40.4%) [15]. However, slightly higher finding has been found in Chitwan, Nepal (56.3%) [19]. Likewise, a higher proportion of the respondents (33.3%) responded poor perineal hygiene followed by having too many children (24.5%) as the risk factor of cervical cancer in this study. A consistent finding has been reported in the study conducted by Devkota et al. [12] where 38.2% reported poor perineal hygiene as a risk factor. However, studies conducted in Nepal by Bharati et al. [18] and Shrestha et al. [20] showed that more than half of the respondents were aware of poor perineal hygiene as a risk factor. Likewise, in a study conducted in Rukum [21], a slightly higher proportion of the respondents reported having too many children as risk factors (45.9%). The least reported risk factor in this study was using oral contraceptives for a long time (6%) which is in line with the study conducted in Nigeria (11.7%) [22]. However, 37.3% of the respondents were not aware of any one of the risk factors in this study which supports the findings by Devkota et al. (31.2%) [12]. Likewise, almost half of the respondents (48.6%) stated lower abdominal pain as signs and symptoms of cervical cancer in this study which is slightly higher than the study conducted in Nigeria (39%) [22] and Nepal (36.4%) [12]. The second most cited sign and symptom in this study was excessive vaginal discharge with an offensive smell (29.7%) which is in line with the study conducted in Bangladesh (25%) [23] and slightly lower than the findings conducted in Nigeria (37.6%) [22]. However, 36.5% were not aware of any one of the signs and symptoms in this study which is as per the study conducted by Devkota et al. (36.4%) [12]. This inconsistency in results between the studies might have emerged from the inadequate knowledge of participants between the countries.

The majority of the respondents (80.3%) answered that cervical cancer is a preventable disease in this study which is lower than the findings conducted in Biratnagar, Nepal, (97.1%) [24] and higher than the findings found in a study by Devkota et al. (64.2%) [12]. In response to preventive measures for cervical cancer in the current study, 35.3% responded maintaining perineal hygiene as one of the measures followed by screening for cervical cancer by 32.5% of the respondents. In contrast to this result, a study conducted by Devkota et al. [12] showed fewer proportions of the respondents' stated perineal hygiene (10.4%) and screening for cervical cancer (13.9%) as preventive measures. The majority (79.1%) of the respondents had not heard about the HPV vaccine and only a few respondents (12.9%) could answer correctly the appropriate time for the HPV vaccine in the present study. The study conducted by Devkota et al. [12] contradicts these findings where 98.3% of the respondents had not heard about the vaccine and only 1.7% knew the appropriate time for the vaccine.

Most of the respondents (71.5%) were not aware of the available screening methods, while 16.9% of the respondents answered Pap smear as one of the screening methods in this study which is similar to the findings conducted by Devkota et al. (21.4%) [12] and contradicts the findings conducted in Biratnagar, Nepal (44.1%) [24]. Similarly, 29.7% of the respondents answered correctly about the ideal age to start cervical cancer screening, whereas a study conducted in Ethiopia [17] showed only 3.6% were aware of it. Only a few respondents (9.6%) answered correctly about the time interval for cervical cancer screening, while the majority (86.7%) of respondents stated cervical cancer is curable if diagnosed in its earliest stage in this study. The study conducted in Biratnagar [24], Nepal, showed that very few were aware of the time interval for screening (1.1%), but most of them knew it is curable if treated early (97.1%). It is of vital importance to be acquainted with the ideal time interval for screening and the importance of follow-up in medical care that can prevent the delay in seeking care which ultimately can lead to timely diagnosis and management. This finding of the study signifies the roles of healthcare providers to focus attention on ideal age, frequency, and follow-up for cervical cancer screening.

In this study, only 38.6% of the respondents had ever been screened for cervical cancer, and the majority (86.5%) of their last time screening was within 5 years. Consistent findings have been reported in a study conducted in Biratnagar, Nepal [24], where 30% had ever been screened for cervical cancer, and the majority (86.9%) of them had been screened within 5 years. A slightly higher finding has been observed in a study conducted by Acharya et al. [25] where 47.6% had ever been screened. In the study conducted in Ethiopia [17], it was found that only 25% had undergone screening. Another study conducted in Nepal [14] and Ghana [16] showed that only 13.6% and 3% of the respondents, respectively, had screened before. Similarly, 37.5% of the respondents stated the reason for performing screening tests was following suggestions from health personnel. This finding suggests that health workers can play a significant role in reducing the burden of cervical cancer disease in the community. Despite the lower proportion of respondents having inadequate knowledge, the results of this study showed relatively higher screening practices. This could be explained by the ongoing cervical cancer prevention program where the study has been conducted. However, this is still lower than the national target coverage which is 50% in women aged 30–60 with recommended screening every five years. Furthermore, health personnel's suggestions regarding screening tests might have led to cervical cancer screening practices in this study context.

Women might face several obstacles or challenges in attending screening programs. Barrier factors might be contextual and could differ concerning specific areas, communities, ethnic groups, or cultures. The top three reasons mentioned in this study regarding barriers to screening included absence of symptoms, unawareness of screening, and feeling of embarrassment which is similar to the findings reported in studies conducted in Nepal [14, 18]. Similarly, a study conducted by Getachew et al. [17] and Shrestha and Dhakal [19] found that the top reason for not undergoing screening was the absence of symptoms. Furthermore, the presence of male doctors, no advice from the healthcare provider, fear of pain, fear of abnormal results, high cost of treatment, presence of male doctors, and lack of time were some other barriers responded to by a few of the respondents in the current study. Therefore, this result highlights the need for an awareness program on cervical cancer screening.

This study showed a statistically significant association between levels of awareness with the age of the respondents (*p* = 0.031), educational level (*p* = 0.013), and number of children (*p* = 0.003). However, no significant association was found with other variables such as age at marriage, ethnicity, occupation, and monthly family income in this study. The study conducted in China [26] showed a similar association between the level of awareness and age of the respondents, whereas the study conducted in Chitwan, Nepal [19], showed no association between the age and level of awareness. Likewise, a significant association was found between the level of awareness and education of respondents in the studies conducted in Nigeria [22] (*p* = 0.005), China [26], and Chitwan, Nepal [19] (*p* = 0.041). However, the study conducted in Ethiopia by Getachew et al. [17] showed no association between the education and level of awareness.

## 5. Conclusion

The study's findings concluded that most of the respondents had inadequate levels of awareness of cervical cancer screening. More than one-third of the respondents had ever been screened for cervical cancer which is still low regarding the national target coverage, and the majority of their last time of screening was within 5 years. The result showed a statistically significant association between levels of awareness with the age of the respondents, educational level, and number of children. The absence of symptoms, unawareness of screening, and feelings of embarrassment were among the most commonly stated barriers to practicing cervical cancer screening among the respondents. Thus, there is a need for awareness about cervical cancer screening among the eligible group considering information on risk factors, signs, and symptoms, the recommended age for screening, its frequencies, and screening procedures used. The result of the study might give an idea to the concerned authority about the existing barriers in the community and plan the intervention strategies for overcoming barriers for maximum utilization of screening in the community setup.

## Figures and Tables

**Table 1:** Sociodemographic characteristics of the respondents.

Variables	Frequency (*n* = 249)	Percent (%)
Age (in years)		
30–39	98	39.4
40–49	94	37.8
≥ 50	57	22.8
Mean ± SD = 42.05 ± 8.37		
Marital status		
Married	235	94.4
Unmarried	6	2.4
Widowed	6	2.4
Divorced	2	0.8
Age at marriage (*n* = 243)		
< 15	14	5.8
15–19	102	42.0
20–24	96	39.5
25–29	29	11.9
≥ 30	2	0.8
Mean ± SD = 19.88 ± 3.743, range in years = 12–35		
Ethnicity		
Brahmin/Chhetri	120	48.2
Janjati	125	50.2
Dalit	4	1.6
Educational level of respondents		
Cannot read and write	72	28.9
Can only read and write	10	4.0
Basic education (1–8)	69	27.7
Secondary (9–12)	79	31.7
University	19	7.7
Occupation of respondents		
Household work	125	50.2
Agriculture	76	30.5
Job holder	5	2.0
Business	43	17.3
Numbers of children (*n* = 240)		
1–2	186	77.5
≥ 3	54	22.5
Average monthly family income (Rs.)		
≤ 40,000	187	75.1
> 40,000	62	24.9

**Table 2:** Awareness of meaning, risk factors, and signs and symptoms of cervical cancer.

Variables	Frequency (*n* = 249)	Percent (%)
Meaning of cervical cancer		
Pain in the cervix	37	14.9
Swelling in the cervix	27	10.8
Abnormal growth of the cells in the cervix^∗^	104	41.8
Wound in the cervix	17	6.8
Do not know	64	25.7
Risk factors^∗∗^		
Early onset of sexual activity	46	18.5
Infection with a sexually transmitted germ/virus (HPV)	27	10.8
Multiple male sexual partners	42	16.9
Smoking cigarettes/tobacco	27	10.8
Grand multiparity/having too many children	61	24.5
Using oral contraceptives for long time	15	6.0
Poor perineal hygiene	83	33.3
Do not know	93	37.3
Signs and symptoms^∗∗^		
Intermenstrual vaginal bleeding	47	18.9
Postmenopausal bleeding	29	11.6
Postcoital vaginal bleeding	22	8.8
Excessive vaginal discharge, often with offensive smell	74	29.7
Lower abdominal pain	121	48.6
Pain in the genital during sexual intercourse	11	4.4
Do not know	91	36.5

^∗^Correct answer.

^∗∗^Multiple response.

**Table 3:** Awareness of preventive measures and screening for cervical cancer.

Variables	Frequency (*n* = 249)	Percent (%)
Cervical cancer is a preventable disease		
Yes^∗^	200	80.3
No	10	4.0
I do not know	39	15.7
Preventive measures for cervical cancer^∗∗^		
Through vaccination of HPV vaccine	26	10.4
Avoid multiple sexual partners	36	14.5
Avoid early sexual intercourse	21	8.4
Avoid having too many children	45	18.1
Avoid using oral contraceptives for long time	22	8.8
Safe sex practice (using condom during sexual intercourse)	23	9.2
Screening for cervical cancer	81	32.5
Maintaining good perineal hygiene	88	35.3
Do not know	80	32.1
Appropriate time for giving HPV vaccine		
Do not know/not heard about HPV vaccine	197	79.1
9–26 years^∗^	32	12.9
After getting sexual exposure	8	3.2
30–44 years	12	4.8
Available screening methods^∗∗^		
Visual acetic acid test (VIA)	28	11.2
Pap smear test	42	16.9
HPV test	10	4.0
Do not know	178	71.5
Ideal time to start cervical cancer screening		
Women immediately after marriage	46	18.5
Elderly women	4	1.6
Women of 20 years and above	61	24.4
Women of 30 years and above^∗^	74	29.7
Time interval for cervical cancer screening		
Once every year	127	51.0
Once in every 5 years^∗^	24	9.6
No need to further test if the first Pap smear test is negative	5	2.0
Do not know	93	37.3
Cervical cancer is curable if diagnosed in its earliest stages		
Yes^∗^	216	86.7
No	6	2.4
Do not know	27	10.8

^∗^Correct answer.

^∗∗^Multiple response.

**Table 4:** Level of awareness on cervical cancer screening.

Variables	Frequency (*n* = 249)	Percent (%)
Adequate	26	10.4
Inadequate	223	89.6

**Table 5:** Women's practices and barriers regarding cervical cancer screening.

Variables	Frequency (*n* = 249)	Percent (%)
Ever been screened for cancer of the cervix		
Yes	96	38.6
No	153	61.4
If yes, last time screened (*n* = 96)		
≤ 5 year	83	86.5
> 5 year	13	13.5
Place of screening test performed (*n* = 96)		
Urban health clinic	15	15.6
Health camp	45	46.9
Private hospital	24	25.0
Government hospital	12	12.5
Reasons for performing the screening test (*n* − 96)		
Personal initiative (routine check-up)	28	29.2
Suggestion from health personnel	36	37.5
Due to gynecological problems	32	33.3
Methods used in screening (*n* = 96)		
Pap smear	37	38.5
Visual inspection with acetic acid	28	29.2
Do not know the methods used	31	32.3
Time interval of screening practice (*n* = 96)		
Once a year	32	33.3
Every 3–5 years	18	18.8
Only if gynecological problem occurs	43	44.8
I cannot remember	3	3.1
Barriers on cervical cancer screening (*n* = 153)		
No presence of symptom	136	54.6
Fear of pain	11	4.4
Fear of abnormal results	4	1.6
High cost of treatment	5	2.0
Feeling of embarrassment	29	11.6
Presence of male doctor	16	6.4
No advice from health care provider	11	4.4
Unaware of screening	44	17.7
Lack of time	2	0.8

**Table 6:** Association between the level of knowledge on cervical cancer screening and selected sociodemographic variables.

Variables	Level of knowledge		*χ* ^2^	*p* value
	Adequate *n* (%)	Inadequate *n* (%)		
Age (in years)				
30–39	16 (16.3)	82 (83.7)	6.934	0.031^∗^
40–49	8 (8.5)	86 (91.5)		
≥ 50	2 (3.5)	55 (96.5)		
Age at marriage				
≥ 20	15 (11.8)	112 (88.2)	0.520	0.471
< 20	11 (9.0)	111 (91.0)		
Ethnicity				
Brahmin/Chhetri	11 (9.2)	109 (90.8)	0.403	0.526
Other than Brahmin/Chhetri	15 (11.6)	114 (88.4)		
Educational level				
Cannot read and write	3 (4.2)	69 (95.8)	8.713	0.013^∗^
Basic education	6 (7.6)	73 (92.4)		
Secondary and above	17 (17.3)	81 (82.7)		
Occupation				
Employed	6 (12.5)	42 (87.5)	0.269	0.604
Unemployed	20 (10.0)	181 (90.0)		
Number of children				
1–2	24 (12.9)	162 (87.1)	7.742	0.003^£∗^
≥ 3	0 (0.0)	54 (100.0)		
Monthly family income				
≤ 40,000	19 (10.2)	168 (89.8)	0.064	0.801
> 40,000	7 (11.3)	55 (88.7)		

^∗^ *p* value is significant at < 0.05 level.

^£^Fisher's exact test.

## Data Availability

The data that support the findings of this study are available from the corresponding author upon reasonable request.
